# Descriptions of two new species and one new subspecies from the *Exocelina okbapensis*-group, and notes on the *E. aipo*-group (Coleoptera, Dytiscidae, Copelatinae)

**DOI:** 10.3897/zookeys.715.15913

**Published:** 2017-11-09

**Authors:** Helena Shaverdo, Bob Sumoked, Michael Balke

**Affiliations:** 1 Naturhistorisches Museum, Burgring 7, 1010 Vienna, Austria; 2 Walian 2, Tomohon Selatan, N Sulawesi 95439, Indonesia; 3 SNSB-Zoologische Staatssammlung München, Münchhausenstraße 21, D-81247 Munich, Germany and GeoBioCenter, Ludwig-Maximilians-University, Munich, Germany

**Keywords:** *Exocelina aipo*-group, *Exocelina okbapensis*-group, Copelatinae, Dytiscidae, new species, New Guinea

## Abstract

Two new species and one new subspecies of *Exocelina* Broun, 1886 from New Guinea are described: *E. okbapensis* Shaverdo & Balke, **sp. n.**, *E. okbapensis hajeki* Shaverdo & Balke, **ssp. n.**, and *E. may* Shaverdo & Balke, **sp. n.** These and two already described species are assigned to the *E. okbapensis*-group, which is morphologically (based on setation of the paramere) and phylogenetically close to the *E. aipo*-group. On the latter, morphological and taxonomic notes are provided. An identification key to all known species of the groups is presented, and important diagnostic characters are illustrated. Data on the species distributions are mapped and show that the species occur only in the central mountain part of the island restricted by Wamena in the west and Sandaun Province in the east.

## Introduction

Here, we continue to build up the infrageneric structure of the genus that was started in our previous taxonomic studies on the New Guinea species of the diving beetle genus *Exocelina* Broun, 1886 (Balke 1998, Shaverdo et al. 2005, 2012, 2016b, c, 2017). A new species group, *E. okbapensis*-group, is proposed for two new species and two already described ones: *E. ketembang* (Balke, 1998) and *E. talaki* (Balke, 1998) based on the shape and setation of the paramere as well as on results of a phylogenetic analysis of molecular data (Toussaint et al. 2014). It is shown that representatives of another species group, namely *E. aipo*-group, share the same shape and setation of the paramere with the new species group and, therefore, both are most likely closely related. This is also supported by a preliminary molecular analysis of the new species and old material of the *E. aipo*-group species and *E. talaki*, which suggests that they form a monophyletic clade.

As in most of our previous papers on the genus, all species data will be presented on the species-id.net portal automatically created by ZooKeys with the publication of this paper (Shaverdo et al. 2012, 2013, 2014, 2016a, b, c, 2017).

## Materials and methods

The present work is based on material from the following collections:

**CGW** Collection of Günther Wewalka, Vienna, Austria

**MZB** Museum Zoologicum Bogoriense, Cibinong, Indonesia

**NHMW** Naturhistorisches Museum Wien, Vienna, Austria

**NMPC** Národní museum, Prague, Czechia

**ZSM** Zoologische Staatsammlung München, Munich, Germany

All methods follow those described in detail in previous articles (Shaverdo and Balke 2014, Shaverdo et al. 2012, 2014). All specimen data are quoted as they appear on the labels attached to the specimens. Label text is cited using quotation marks. Comments in square brackets are ours. The following abbreviations were used:

**TL** total body length

**TL-H** total body length without head

**MW** maximum body width

**hw** handwritten

## Notes on diagnostic characters of the groups

As already mentioned above, both groups build a monophyletic clade according to the results of molecular analyses (Toussaint et al. 2014; unpublished results). This monophyly is also supported morphologically by the structure and setation of the male genitalia, especially of the paramere. The paramere of all representatives of the groups have a distinct notch on its dorsal side and a small, evidently separated subdistal part with a tuft of very dense, strong setae; proximal setae of the paramere are sparse and fine, inconspicuous (e.g., Fig. 5C; figs 32–35 in Balke (1998)). Other diagnostic characters of the clade comprising these two groups are:

– beetles small or middle-sized (TL-H 3.25–4.65 mm);

– habitus oblong-oval (broadest approximately at elytral midlength), with rounded pronotal and elytral sides, body outline continuous;

– pronotum short, trapezoidal, with posterior angles not drawn backwards;

– coloration brown to piceous, mainly uniform, sometimes with paler head and pronotum and darker elytra;

– microreticulation and punctation of dorsal surface very fine to strongly impressed, beetles shiny to matt dorsally;

– metacoxae and abdominal ventrites 1–5 (and 6 in males) with thin, almost longitudinal striae/strioles;

– pronotum and elytra without striae or strioles;

– pronotum with lateral bead;

– antennomeres not modified or modified: mainly, antennomeres 3–6 strongly or slightly enlarged in male and stout in female; antennomere 2 elongate;

– male protarsomeres 1–3 not expanded laterally;

– male protarsomere 4 cylindrical, narrow, with large or small anterolateral hook-like seta;

– median lobe of aedeagus with continuous outline in ventral and lateral view;

– ventral sclerite of median lobe more or less deeply divided apically;

– median lobe without setation.

**Figure 1.**
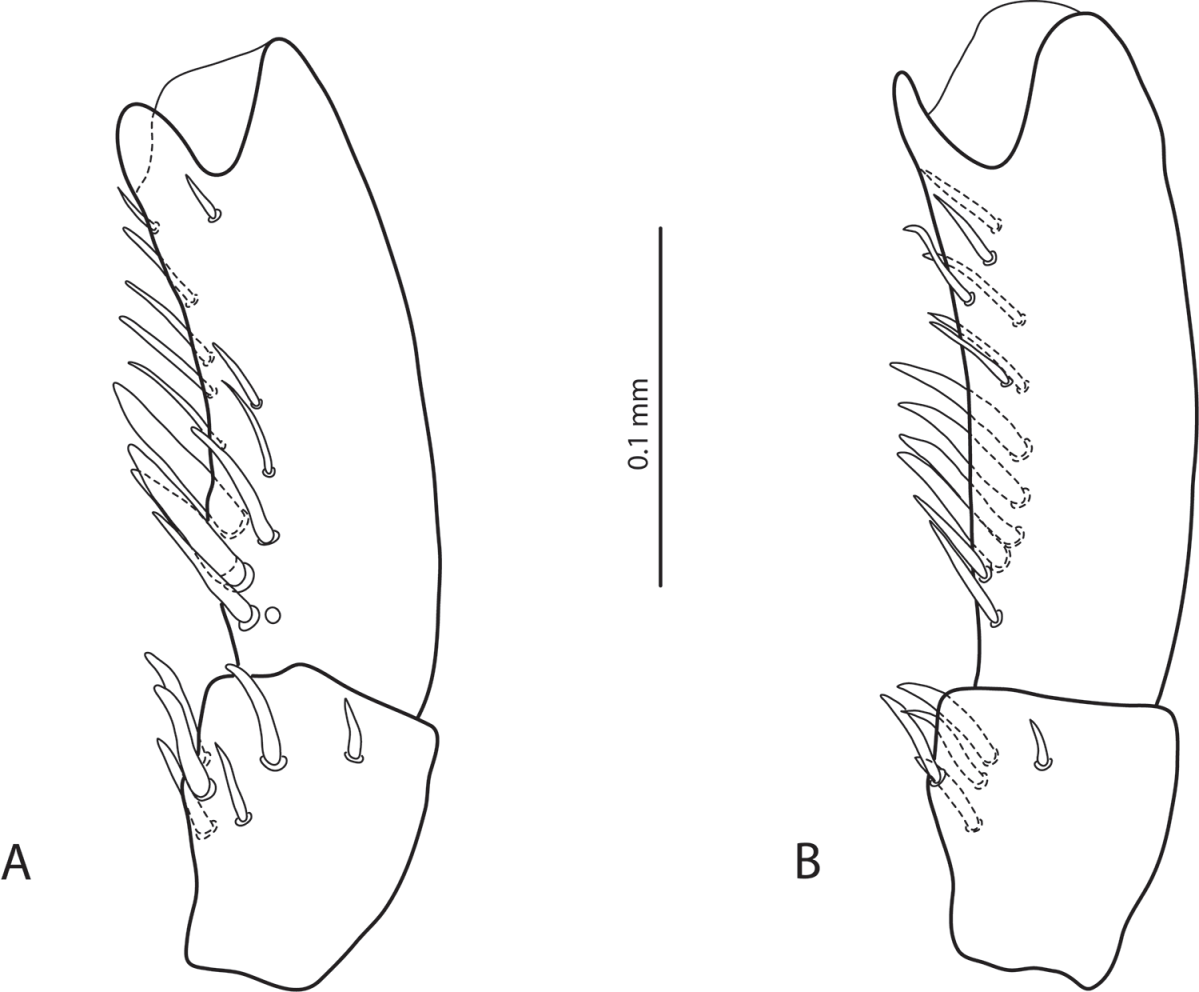
*Exocelina manfredi* (Balke, 1998), male protarsomeres 4–5 in lateral view **A** paratype, Borme **B** Aipomek-Takime.

Representatives of the two groups can be distinguished by the shape and setation of the male protarsomere 5. The species of the *E. aipo*-group have distinctly modified male protarsomere 5: concave ventrally, usually with some ventral setae enlarged and shifted to base (Fig. 1; figs 25–28 in Balke (1998)), whereas the species of the *E. okbapensis*-group have the male protarsomere 5 long and narrow, without concavity, ventrally with two rows of relatively short setae (e.g., Fig. 5D).

## Checklist and distribution of the species of the *Exocelina aipo*- and *okbapensis*-groups

Abbreviations: IN – Indonesia, PNG – Papua New Guinea.

**Table:** 

	*Exocelina aipo*-group	
1.	*Exocelina aipo* (Balke, 1998)	IN: Papua: Yahukimo
2.	*Exocelina karmurensis* (Balke, 1998)	IN: Papua: Yahukimo
3.	*Exocelina manfredi* (Balke, 1998)	IN: Papua: Pegunungan Bintang
4.	*Exocelina me* (Balke, 1998)	IN: Papua: Pegunungan Bintang
	***Exocelina okbapensis*-group**	
1.	*Exocelina okbapensis* sp. n.	IN: Papua: Pegunungan Bintang; PNG: Sandaun
1a.	*Exocelina okbapensis hajeki* ssp. n.	IN: Papua: Jayawijaya
2.	*Exocelina may* sp. n.	PNG: Sandaun
3.	*Exocelina ketembang* (Balke, 1998)	IN: Papua: Yahukimo, Pegunungan Bintang
4.	*Exocelina talaki* (Balke, 1998)	IN: Papua: Pegunungan Bintang

## Species descriptions

### *Exocelina aipo*-group

The representatives of the group have male protarsomere 5 distinctly modified: it is concave ventrally, with some ventral setae enlarged and shifted to base, except those in *E. karmurensis*. They also have very similar shape of the median lobe of the aedeagus: in lateral view, it is curved, with apex curved downwards and slightly rounded; in ventral view, it is evenly tapering to the broadly pointed apex (fig. 40 in Balke 1998). The most evident difference is its size: the shortest medial lobe is in *E. manfredi* and the longest and most robust one is in *E. karmurensis*. Shape of the paramere is also very similar: it has a distinct notch on dorsal side and a small, evidently separated subdistal part with a tuft of very dense, strong setae; proximal setae inconspicuous (figs 32–35 in Balke 1998).

To date, only four species of the group are described and no new species have been discovered. The only possible exception is one male from Aipomek-Tanime area (“IR 92#17a: West New Guinea, Aipomek-Tanime, 2000 m, 20.viii.1993, Balke” (NHMW)), which was mentioned in Balke (1998) under sp.5. Most likely, it belongs to *E. manfredi*, which was described from Borme area. The only morphological difference is less modified male protarsomere 5: concavity very shallow and basal enlarged setae smaller than in the type specimens from Borme (Fig. 1). Additional material is requited for a conclusion whether it is a form of *E. manfredi* or a new species of the *E. aipo*-group.

The differences between the species of this group are given in the key. For their descriptions, see Balke (1998).

### *Exocelina okbapensis*-group

#### 1. Exocelina okbapensis

Taxon classificationAnimaliaORDOFAMILIA

Shaverdo & Balke sp. n.

http://zoobank.org/FD15BCDA-16A7-4961-B4A7-E44F2AEB0DAF

Figs 2 , 5

Sp. 4: Balke 1998: 338.

##### Type locality.

Papua: Pegunungan Bintang Regency, Ok Bap, 4°49'28.6"S; 140°24'47.0"E, 1961 m a.s.l.

##### Type material.

*Holotype*: male “Indonesia: Papua, nr Ok Bab [sic!], 1961m, 8.vi.2015, -4,82460033148527 140.413050251081, Sumoked (Pap049)” (MZB). *Paratypes*: **Indonesia: Papua**: 10 males and 6 females with the same label as the holotype (MZB, NHMW, ZSM). 3 males, 3 females “Indonesia: Papua, nr Ok Bab, 2121 m, 8.vi.2015, -4,84880341216921 140,367602147161, Sumoked (Pap050)”, one male and one female with additional labels with green text “M. Balke 7002” and “M. Balke 7003” (NHMW, ZSM). 3 males, 2 females “Indonesia: Papua, N Ok Sibil, 1564m, 10.vi.2015, -4,87003368 140,64401, Sumoked (Pap052)”, two males with additional labels with green text “M. Balke 7000” and “M. Balke 6999” (NHMW, ZSM). **PNG: Sandaun**: 1 male “PAPUA, Selminumtem [Selminum Tem, 45 km SWS Telefomin, ca. 5°S; 141°15'E], W.Sepik d. P.Beron leg.”, “Copelatus nomax J.B.Br. det.V. Guéorguiev 1917” [partly hw] (NHMW). 1 male “PAPUA N.G.: Sandaun Prov. Telefomin, 16.-17.5.1998 trail to Eliptamin 1700–1800 m; leg. Riedel” (NHMW).

##### Diagnosis.

Beetle medium-sized; usually piceous, with brown pronotal sides; more or less shiny to submatt; pronotum with distinct lateral bead; antennomeres simple or stout; male protarsomere 4 with large, thick, strongly curved anterolateral hook-like seta; male protarsomere 5 long and narrow, without concavity, with anterior row of 18–27 and posterior row of eight relatively short setae; median lobe curved, with apex curved downwards and slightly rounded in lateral view. The species is similar to *E. me* but its protarsomere 5 is not modified. From *E. may* sp. n., it differs in a large, strongly curved anterolateral hook-like seta of the male protarsomere 4.

##### Description.

**Figures 2–4.**
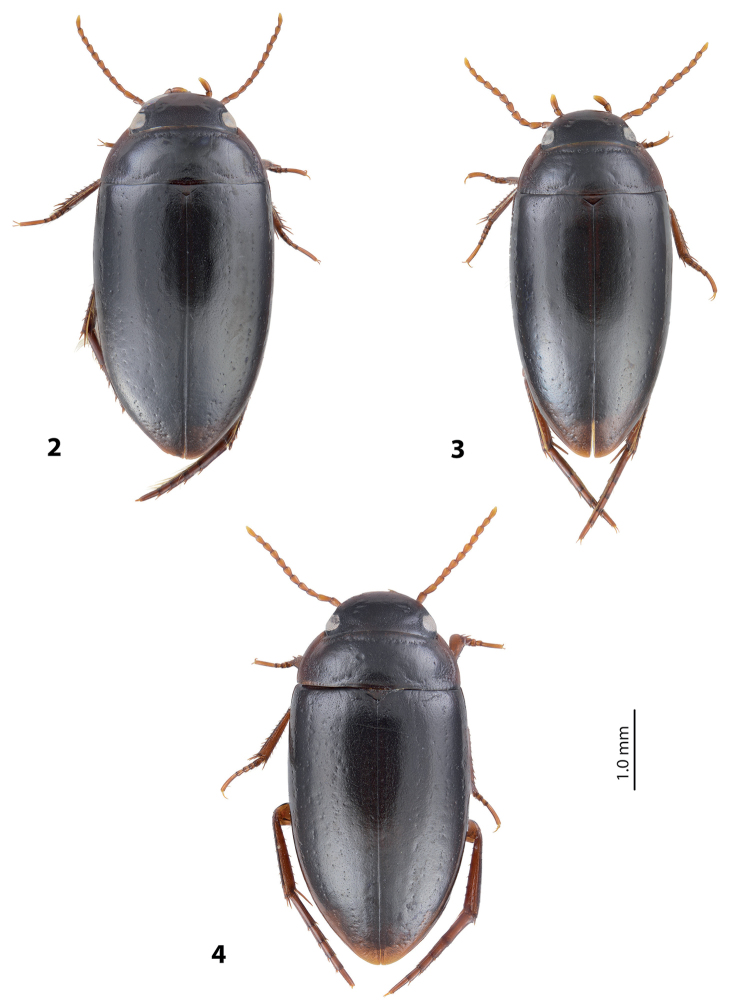
Habitus and coloration **2** *Exocelina okbapensis* sp. n. **3** *E. okbapensis hajeki* ssp. n. **4** *E. may* sp. n.

*Size and shape*: Beetle small to medium-sized (TL-H 3.35–4.5 mm, TL 3.7–4.9 mm, MW 1.75–2.4 mm for Papua populations), with oblong-oval habitus, broadest at elytral middle. Medium-sized specimens more common. *Coloration*: Head, disc of pronotum and elytra dark brown to piceous, pronotal sides broadly reddish brown, some specimens with narrow reddish sutural lines; head appendages and legs reddish brown, distally darker (Fig. 2). Teneral specimens paler.

*Surface sculpture*: Head with dense punctation (spaces between punctures 1–2 times size of punctures), finer and sparser anteriorly; diameter of punctures equal to diameter of cells of microreticulation. Pronotum and elytra with distinct punctation, sparser and finer than on head. Elytral punctation slightly sparser than pronotal one. Pronotum and elytra with evident microreticulation, dorsal surface more or less shiny to submatt. Head with microreticulation stronger. Metaventrite and metacoxa distinctly microreticulate, metacoxal plates with longitudinal strioles and transverse wrinkles. Abdominal ventrites with distinct microreticulation, strioles, and very fine sparse punctation.

*Structures*: Pronotum with distinct lateral bead. Base of prosternum and neck of prosternal process with distinct ridge, slightly rounded anteriorly. Blade of prosternal process lanceolate, relatively broad, slightly convex, with distinct lateral bead and few setae; neck and blade of prosternal process evenly joined. Abdominal ventrite 6 broadly rounded.

**Figure 5.**
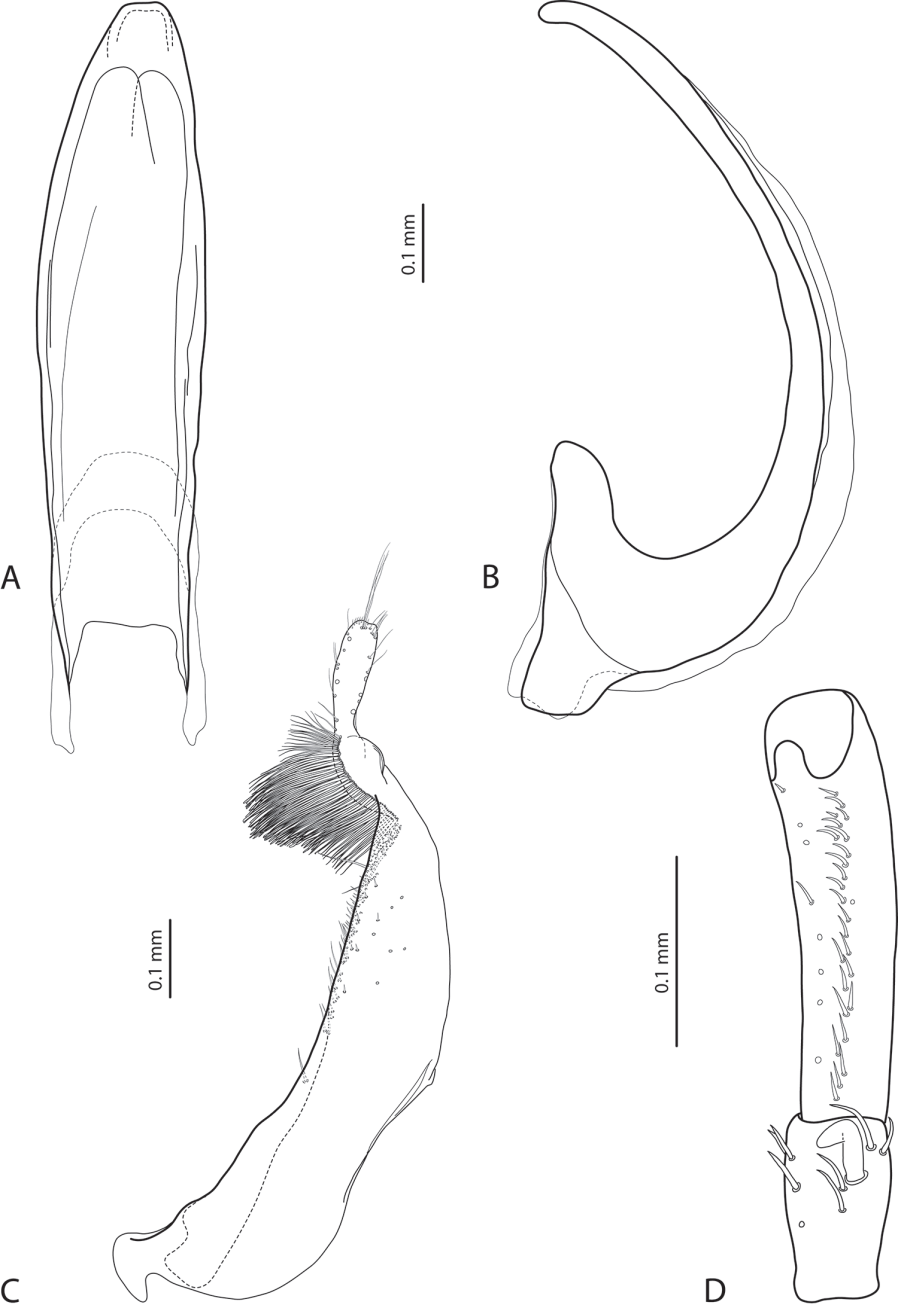
*Exocelina okbapensis* sp. n. **A** median lobe in ventral view **B** median lobe in lateral view **C** paramere in external view **D** male protarsomeres 4–5 in ventral view.

*Male*: Antennae simple or stout (Fig. 2). Protarsomere 4 with large, thick, strongly curved anterolateral hook-like seta. Protarsomere 5 ventrally with anterior row of 18–27 and posterior row of 8 relatively short setae (Fig. 5D). Median lobe curved, with apex curved downwards and slightly rounded in lateral view and evenly tapering to the broadly pointed apex in ventral view. Paramere with distinct notch on its dorsal side and small, evidently separated subdistal part with a tuft of very dense, strong setae; proximal setae inconspicuous, strongly reduced in some specimens (Fig. 5A–C). Abdominal ventrite 6 with 5–16 long lateral striae on each side.

*Holotype*: TL-H 4.2 mm, TL 4.6 mm, MW 2.25 mm.

*Female*: Antennae simple. Pro- and mesotarsi not modified. Abdominal ventrite 6 without striae.

##### Variability.

In some males, antennomeres 2–6 slightly thicker, stout. Some specimens have the dorsal microreticulation more strongly impressed. Two specimens from Sandaun are smaller than most of the Papua specimens (TL-H 3.3–3.65 mm, TL 3.7–4.1 mm, MW 1.75–1.95 mm) and have less dense subdistal setae on the paramere.

##### Distribution.

Papua: Pegunungan Bintang Regency and Papua New Guinea: Sandaun Province (Fig. 12).

##### Habitat.

Near Ok Bap, the species was collected in small creeks as well as in slowly flowing, sun exposed irrigation ditches along road.

##### Etymology.

The species is named after Ok Bap, where most of the specimens were collected. The name is an adjective in the nominative singular.

#### 1a. Exocelina okbapensis hajeki

Taxon classificationAnimaliaORDOFAMILIA

Shaverdo & Balke ssp. n.

http://zoobank.org/56F86E0C-984E-4066-92FB-4739CD8DD7EC

Figs 3 , 6

Exocelina undescribed sp. MB0066: Toussaint et al. 2014: Supplementary figs 1–4, tab. 2.

##### Type locality.

Papua: Jayawijaya Regency, Wamena, 04°03.6'S; 139°01.9'E, 2050 m a.s.l.

##### Type material.

*Holotype*: male “IN, PA: Jayawijaya Regen., Baliem vall., 10km NE Wamena, forest above ‘Baliem vall. Resort’, 2050 m, 2–3.II.2015, 04°03.6'S, 139°01.9'E; J.Hájek & J.Šumpich leg” (NMPC). *Paratypes*: 2 males and 7 females with the same label as the holotype, one female with an additional label “M. Balke 7372” (MZB, NHMW, NMPC, ZSM). 6 males, 11 females “INDONESIA, Papua: Jayawijaya Distr., Ballem valley, 10km NE of Wamena, forest above “Baliem valley resort”, 04°03.6'S, 139°01.9'E, 2050 m; 2-3.ii.2015 J.Hájek & J.Šumpich leg.” (NHMW, NMPC, ZSM). 1 male “66 M. Balke” [green], “measured J. Parkin 43”, “Indonesia: Irian Jaya, N Wamena, Cerny, M. Balke: MB 66” (ZSM).

##### Diagnosis.

Beetle medium-sized; dark brown to piceous, with reddish brown pronotal sides; submatt; pronotum with distinct lateral bead; male antennomeres 2–6 slightly, but evidently enlarged, female antennomeres 2–6 stout; male protarsomere 4 with large, thick, strongly curved anterolateral hook-like seta; male protarsomere 5 long and narrow, without concavity, with anterior row of 26 and posterior row of eight relatively short setae; median lobe curved, with apex curved downwards and slightly rounded in lateral view. The subspecies differs from the nominative subspecies in the modified antennae, in the shape and setation of the paramere, and in the more striated abdominal ventrite 6; some beetles are also somehow slightly more matt due to more strongly impressed dorsal microreticulation.

##### Description.

*Size and shape*: Beetle medium-sized (TL-H 3.75–4.5 mm, TL 4.1–4.9 mm, MW 2.0–2.3 mm), with oblong-oval habitus, broadest at elytral middle (Fig. 3). *Coloration*: As in nominative subspecies.

*Surface sculpture*: As in nominative subspecies apart from pronotum and elytra with evident, rather strongly impressed microreticulation, dorsal surface submatt.

*Structures*: As in nominative subspecies.

**Figure 6.**
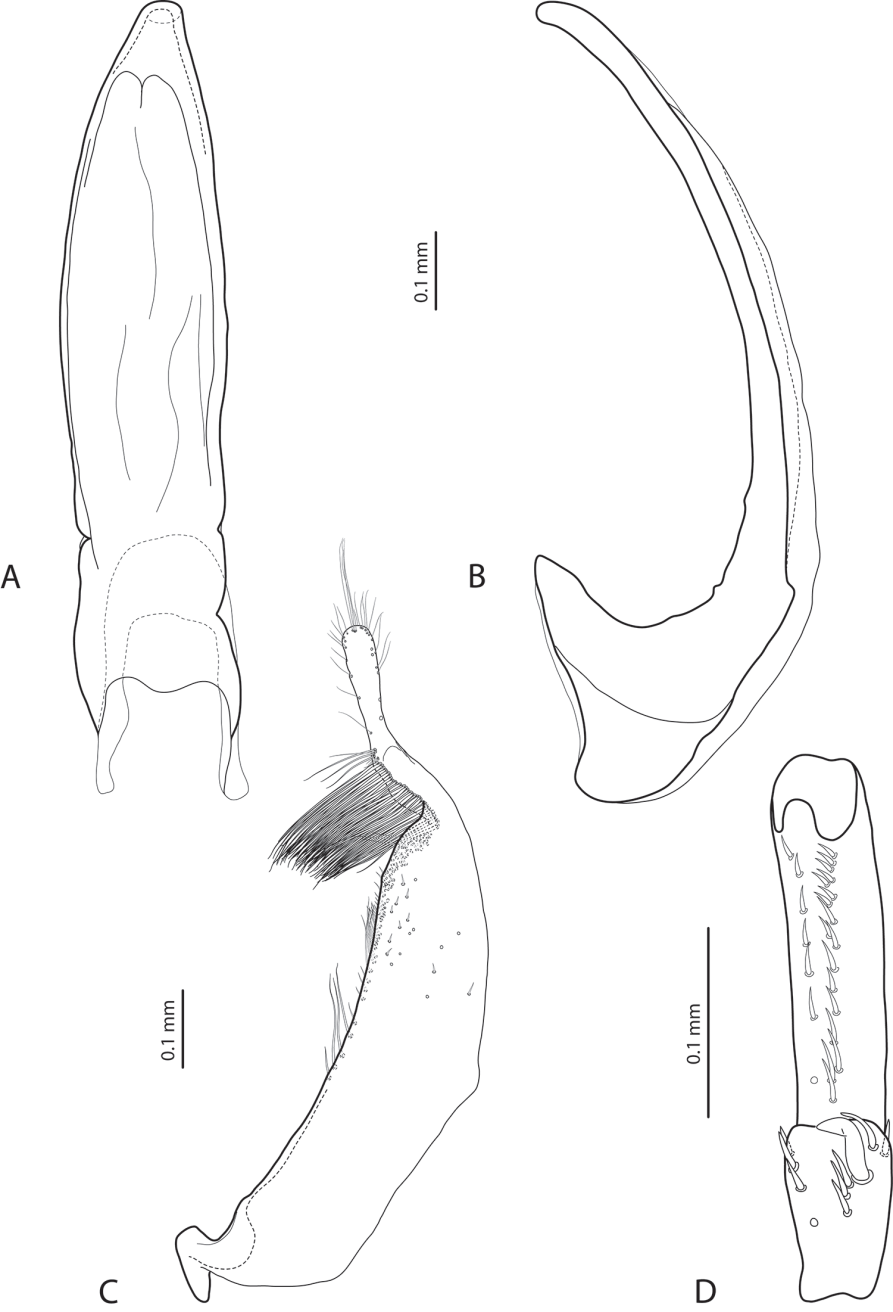
*Exocelina okbapensis hajeki* ssp. n. **A** median lobe in ventral view **B** median lobe in lateral view **C** paramere in external view **D** male protarsomeres 4–5 in ventral view.

*Male*: Antennomeres 2–6 slightly, but evidently enlarged, antennomere 2 with slightly extended external upper angle (Fig. 3). Protarsomere 4 with large, thick, strongly curved anterolateral hook-like seta. Protarsomere 5 ventrally with anterior row of 26 and posterior row of eight relatively short setae (Fig. 6D). Median lobe curved, with apex curved downwards and slightly rounded in lateral view and evenly tapering to the slightly truncate apex in ventral view. Paramere with distinct notch on dorsal side and small, evidently separated subdistal part with a tuft of very dense, strong setae; proximal setae inconspicuous (Fig. 6A–C). Abdominal ventrite 6 with numerous (16–22) long lateral striae on each side.

*Holotype*: TL-H 4.0 mm, TL 4.4 mm, MW 2.05 mm.

*Female*: Antennomeres 2–6 stout, in some specimens only slightly more slender than in males, antennomere 2 with slightly extended external upper angle; pro- and mesotarsi not modified and abdominal ventrite 6 without striae.

##### Variability.

In some males, antennomeres 2–6 more strongly enlarged, in some others less strongly enlarged, similar to those of females.

##### Distribution.

Papua: Jayawijaya Regency. The subspecies is known only from the type locality (Fig. 12).

##### Habitat.

Near Wamena, the species was collected from wet ground with weak water flow and forest puddles, which turn to a small creek during rain (Figs 13–14).

##### Etymology.

The subspecies is named after our friend and colleague Jirí Hájek who collected almost all the specimens. The name is a noun in the genitive case.

#### 2. Exocelina may

Taxon classificationAnimaliaORDOFAMILIA

Shaverdo & Balke sp. n.

http://zoobank.org/C403A69C-67D8-49AB-A65B-1A4D3BD166B9

Figs 4 , 7

Exocelina undescribed sp. MB0662: Toussaint et al. 2014: Supplementary figs 1–4, tab. 2.
Exocelina undescribed sp. MB0671: Toussaint et al. 2014: Supplementary figs 1–4, tab. 2.

##### Type locality.

Papua New Guinea: Sandaun Province, May River, 04°49.80'S; 141°38.17'E, above 2000 m a.s.l.

##### Type material.

*Holotype*: male “M. Balke 662”, “Papua New Guinea: Sandaun, May River (WB47), 15.x.2003, K. Sagata, M. Balke: MB 662” (ZSM). *Paratypes*: 1 male, 2 females “Papua New Guinea: Sandaun, May River, 8.x.2003, 2627m, 4 49.779S 141 38.174E K. Sagata (WB47)”, male with an additional label “M. Balke 663” (ZSM). 1 male “M. Balke 664”, “Papua New Guinea: Sandaun: Sandaun, May River (WB47), 15.x.2003, K. Sagata, M. Balke: MB 664” (ZSM).

##### Additional material.

1 male “M. Balke 671”, “Papua New Guinea: Sandaun, Mekil (WB106), 14.x.2003, K. Sagata, M. Balke: MB 671” (ZSM). 1 male “M. Balke 679”, “Papua New Guinea: Sandaun, Mekil (WB106), 14.x.2003, K. Sagata, M, Balke: MB 679” (ZSM).

##### Diagnosis.

Beetle medium-sized; piceous, with brown head and sides of pronotum; submatt, with distinct punctation and microreticulation; male antennae simple; male protarsomere 4 with small (smaller than more laterally situated large seta), weakly curved anterolateral “hook-like” seta; male protarsomere 5 long and narrow, without concavity, with anterior and posterior rows of relatively short setae; median lobe curved, with apex curved downwards and slightly rounded in lateral view. The species is similar to the submatt representatives of the *E. aipo*-group with non-modified antennae but differs from them in non-modified male protarsomere 5. From *E. okbapensis* sp. n., it differs in the small and weakly curved anterolateral “hook-like” seta of the male protarsomere 4.

##### Description.

*Size and shape*: Beetle medium-sized (TL-H 3.6–4.65 mm, TL 3.75–5.1 mm, MW 1.9–2.4 mm), with oblong-oval habitus, broadest at elytral middle. *Coloration*: Head, pronotum and elytra dark brown to piceous, pronotal sides sometimes reddish brown; head appendages and legs reddish brown, distally darker (Fig. 4).

*Surface sculpture*: Head with relatively dense and coarse punctation (spaces between punctures 1–2 times size of punctures); diameter of punctures equal to diameter of cells of microreticulation. Pronotum with finer, sparser punctation, and more evenly distributed punctation than on head. Elytra with sparser punctation than on pronotum. Pronotum and elytra with distinct microreticulation, dorsal surface submatt. Head with microreticulation slightly stronger. Metaventrite, metacoxa, and abdominal ventrites distinctly microreticulate. Metacoxal plates with longitudinal strioles and transverse wrinkles; abdominal ventrites with strioles. Ventrum with inconspicuous punctation, more evident on metacoxal plates and two last abdominal ventrites.

*Structures*: Pronotum with distinct lateral bead. Base of prosternum and neck of prosternal process with distinct ridge, slightly rounded anteriorly. Blade of prosternal process lanceolate, relatively broad, slightly convex and smooth, with distinct lateral bead and few lateral setae; neck and blade of prosternal process evenly joined. Abdominal ventrite 6 broadly rounded.

**Figure 7.**
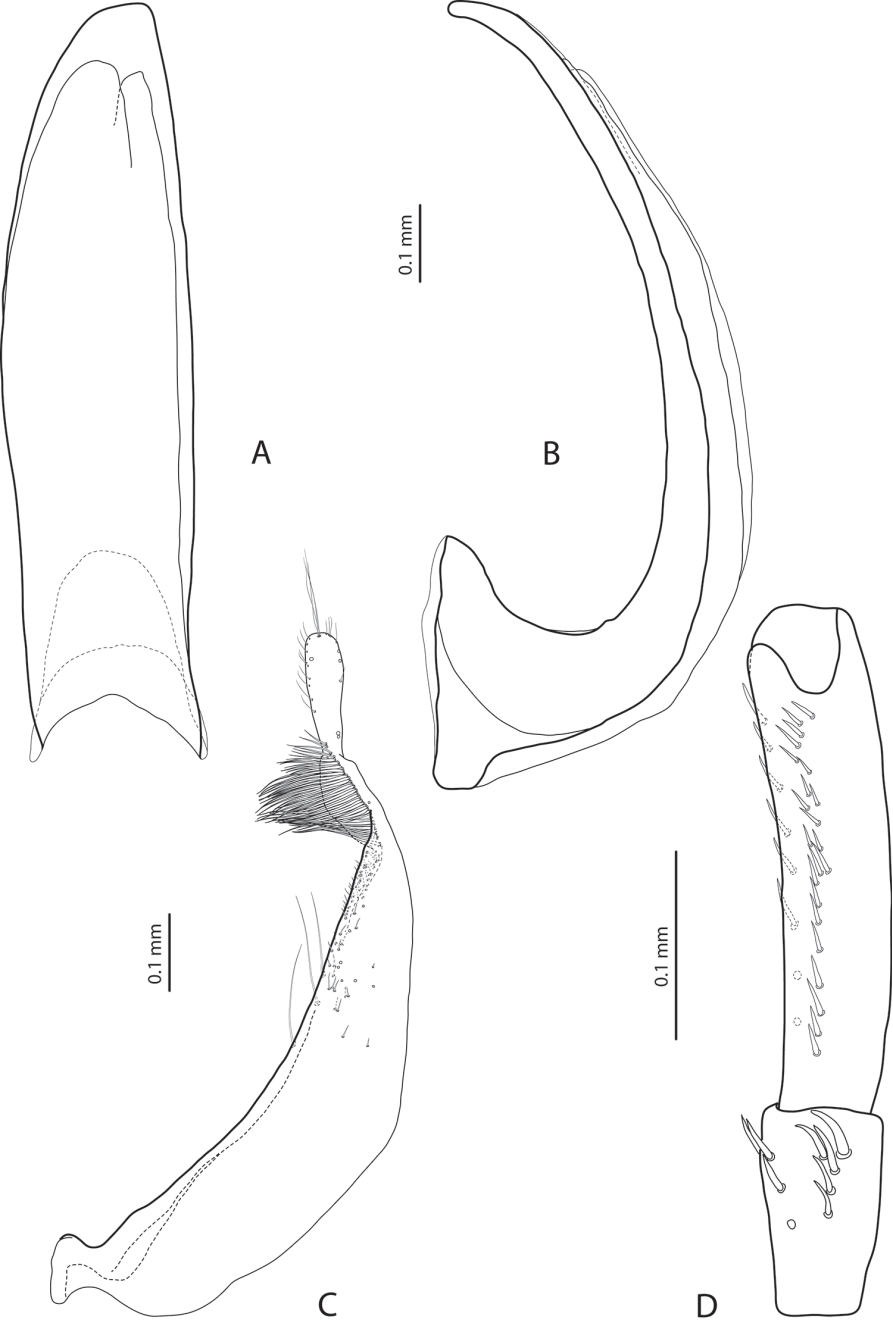
*Exocelina may* sp. n. **A** median lobe in ventral view **B** median lobe in lateral view **C** paramere in external view **D** male protarsomeres 4–5 in ventral view.

*Male*: Antennae simple (Fig. 4). Protarsomere 4 with small (smaller than more laterally situated large seta), weakly curved anterolateral “hook-like” seta. Protarsomere 5 long and narrow, without concavity, ventrally with anterior row of 14–22 and posterior row of 4–9 relatively short setae (Fig. 7D). Median lobe curved, with apex curved downwards and slightly rounded in lateral view. Paramere with distinct notch on its dorsal side and small, evidently separated subdistal part with a tuft of very dense, strong setae; proximal setae inconspicuous (Fig. 7A–C). Abdominal ventrite 6 with 4–9 lateral striae on each side.

*Holotype*: TL-H 4.25 mm, TL 4.7 mm, MW 2.15 mm.

*Female*: Without evident differences in external morphology from males, except for non-modified pro- and mesotarsi and abdominal ventrite 6 without striae.

##### Variability.

Two males from Mekil have dorsal punctation finer and microreticulation less strongly impressed, therefore, they are shinier. Shape and setation of the genitals are very similar to those of the May specimens but also difficult to estimate precisely since both beetles are teneral. For these reasons, they are considered to belong to the species but are not included into the type series.

##### Distribution.

Papua New Guinea: Sandaun Province. The species is known from two localities in the southern part of the province (Fig. 12).

##### Etymology.

The species is named after May River. The name is a noun in the nominative singular standing in apposition.

#### 3. Exocelina ketembang

Taxon classificationAnimaliaORDOFAMILIA

(Balke, 1998)

Figs 8 , 10

Copelatus (Papuadytes) ketembang Balke, 1998: 332; Nilsson 2001: 77 (catalogue).
Papuadytes ketembang (Balke, 1998): Nilsson and Fery 2006: 56 (comb.n.).
Exocelina ketembang (Balke, 1998): Nilsson 2007: 34 (comb.n.).
Exocelina ketembang (Balke, 1998): MB0680: Toussaint et al. 2014: Supplementary figs 1–4, tab. 2.

##### Type locality.

Papua: Pegunungan Bintang Regency, Borme, according to the original description.

##### Type material studied.

*Holotype*: The holotype and 46 paratypes from the same locality have not been found. According to the original description (Balke 1998), they were deposited at the NHMW and had labels with the following data: Irian Jaya, Jayawijaya Province, Borme, 900 m, 18.viii.1992, IR 92#17, M. Balke leg. No beetles with such label data have been found in the collections of NHMW, ZSM, CGW, or in London. Moreover, the label data of this locality (17 from 1992) are wrongly given in the original descriptions not only for this species but also for *Copelatus takime* Balke, 1998: locality 17 is not Borme but Kali Takime, as the labels of the type specimens of *C. takime* state. There are four males (three in NHMW and one in CGW) with the geographical labels “IRIAN JAYA Zentralmassive 140°25'E, 04°24'S" and “14./17.viii.1992 Borme, 1900 m leg. Balke (11)”, one of which has a red holotype label. Therefore, perhaps, the holotype label was simply misquoted in the original description. However, since the type series, indicated in the original description, is very large (47 specimens), investigations on this subject are ongoing.

*Paratypes*: 2 males “IRIAN JAYA: Borme, Tarmlu, 1500 m 6.9.1993”, “ca. 140°25'E, 04°24'S leg. M. Balke (4)”, “Paratypus Copelatus ketembang Balke des. 1997” [red], one of them with an additional green label “M. Balke 3284” (NHMW). 3 males “IRIAN JAYA: Borme, Tarmlu, 1500 m, 6.ix.1993”, “ca. 140°25'E, 04°24'S leg. M. Balke (4-6)”, “Paratypus Copelatus ketembang Balke des. 1997” [red], one of them with two additional labels “M. Balke 3285” [green] and “M. Balke 6410” [green text] (NHMW). 4 males “IRIAN JAYA Zentralmassive 140°25'E, 04°24'S", “14./17.8.1992 Borme, 1900 m leg. Balke (11)”, “Paratypus Copelatus ketembang Balke des. 1997” [red] (CGW, NHMW). 1 male “IRIAN JAYA: 1.x.1993 Eme Gebiet Okloma, 1500 m”, “ca. 139°55'E, 04°14'S leg. M. Balke (28)”, “Paratypus Copelatus ketembang Balke des. 1997” [red] (NHMW). 5 males, 4 females “IRIAN JAYA, 7.9.1992 Kono, 1800 m 139°47'E, 04°21'S, leg. Balke (41)” (CGW, NHMW); the females might belong to two species, *Exocelina ketembang* and *E. damantiensis* (Balke, 1998).

##### Additional material.

**Indonesia: Papua Province: Pegunungan Bintang Regency**: 1 female “IRIAN JAYA Zentralmassive 140°25'E, 04°24'S", “14./17.8.1992 Borme, 1900 m leg. Balke (11)” (NHMW). 1 male, 1 female “IRIAN JAYA Zentralmassive 140°25'E, 04°24'S", “Borme, 1800 m 16.8.1992 leg. Balke (12, 12 A)” (NHMW). **Papua New Guinea: Sandaun Province**: 1 male “M. Balke 670”, “Papua New Guinea: Sandaun, Mekil (WB106), 14.x.2003, K.Sagata, M. Balke: MB 670” (ZSM). 1 male “M. Balke 680”, “Papua New Guinea: Sandaun, Mekil (WB106), 14.x.2003, K. Sagata, M. Balke: MB 680” [04°48.742S 141.39.075E] (ZSM). 1 male “M. Balke 3727” [green], “Papua New Guinea: Sandaun, Ofektaman, 820 m, 17.x.2008, 5.04.113S 141.35.841E, Ibalim (PNG 190)” (ZSM).

##### Females of doubtful identity.

**Indonesia: Papua Province: Pegunungan Bintang Regency**: 1 female “IR 92#17B: West New Guinea, Tanime, 1600 m, 20.viii.1992, Balke” (NHMW); this female might belong to three species: *Exocelina ketembang*, *E. danae* (Balke, 1998) and *E. damantiensis*. 1 female “IRIAN JAYA, 7.ix.1992 Kono, 1800m 139°47'E, 04°21'S, leg. Balke (41) (CGW), the female might belong to two species: *Exocelina ketembang* and *E. damantiensis*. **Papua New Guinea: Sandaun Province**: 3 females “Papua New Guinea: Sandaun, MekilK [sic!], 1718 m, 14.x.2003, 4 48.742S 141 39.075E, K. Sagata (WB 106)” (ZSM); these females might belong to two species: *E. sandaunensis* Shaverdo & Balke, 2014 and *E. ketembang*. 7 females “Papua New Guinea: Sandaun, Ofektaman, 820 m, 17.x.2008, 5.04.113S 141.35.841E, Ibalim (PNG 190)” (ZSM); these females might belong to three species: *E. sandaunensis*, *E. aipomek* (Balke, 1998), and *E. ketembang*.

##### Diagnosis.

**Figures 8–9.**
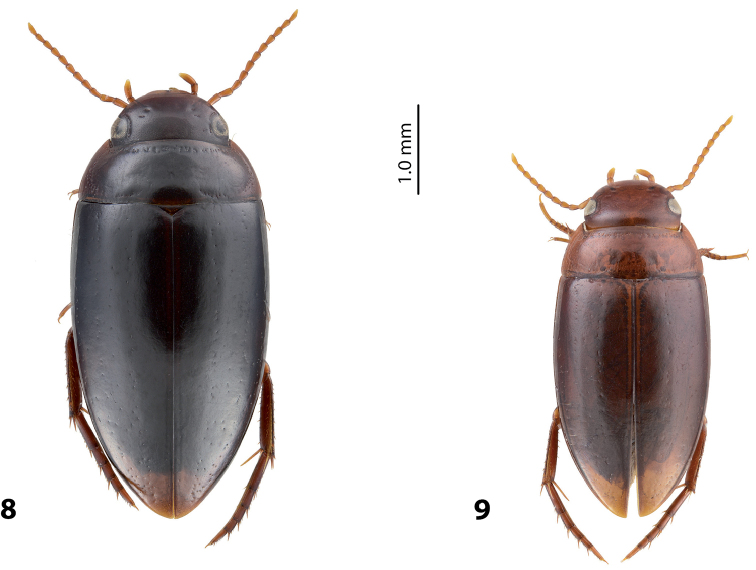
Habitus and coloration **8** *Exocelina ketembang* (Balke, 1998) **9** *E. talaki* (Balke, 1998).

**Figures 10–11.**
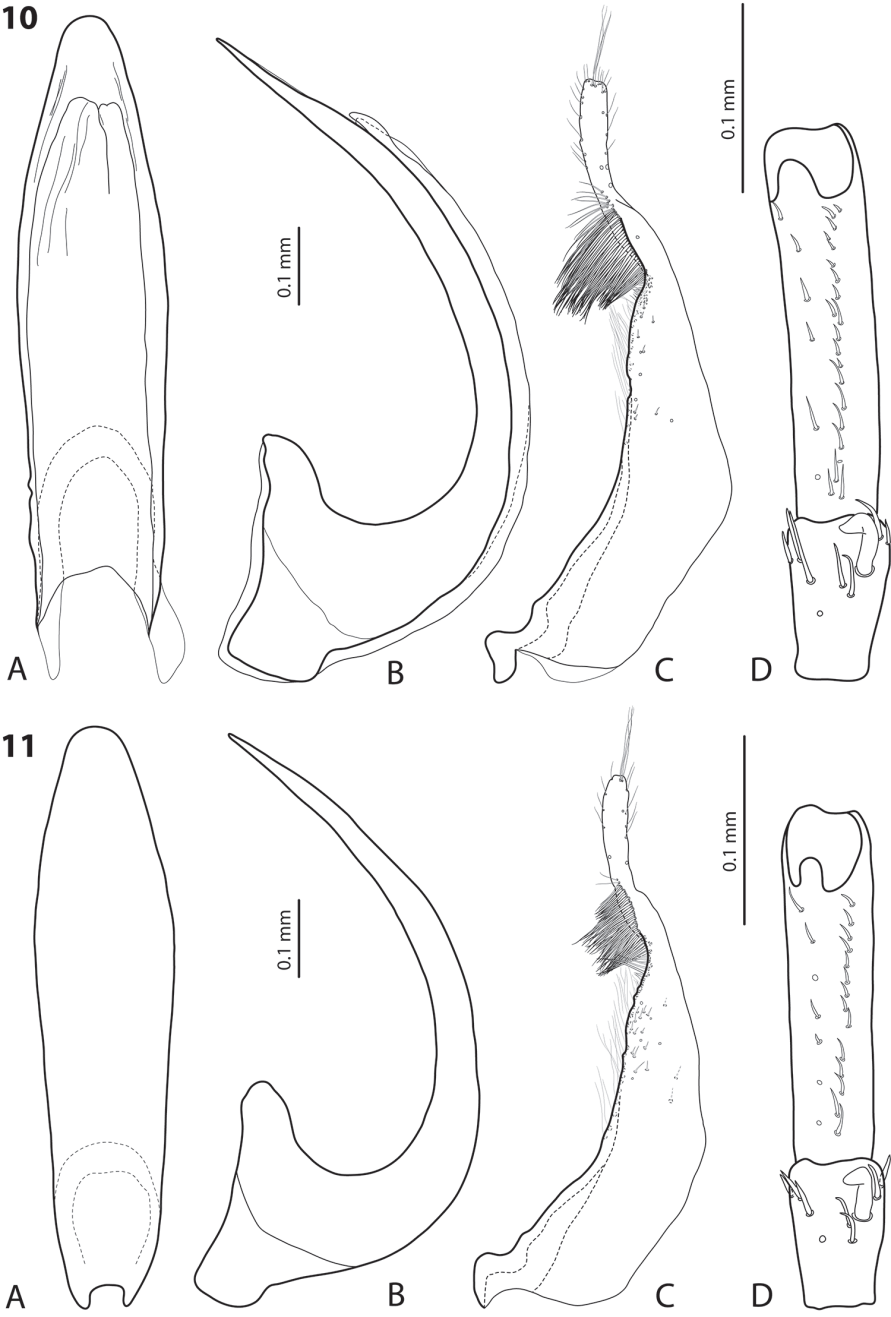
**10** *Exocelina ketembang* (Balke, 1998) **11** *E. talaki* (Balke, 1998) **A** median lobe in ventral view **B** median lobe in lateral view **C** paramere in external view **D** male protarsomeres 4–5 in ventral view.

Beetle medium-sized (TL-H 3.9–4.35 mm); oblong-oval; dark brown to piceous, sometimes with reddish brown pronotal sites and head anteriorly; shiny, with very fine punctation and weakly impressed microreticulation; pronotum with distinct lateral bead; male antennae simple (Fig. 8); male protarsomere 4 with large, thick, strongly curved anterolateral hook-like seta; male protarsomere 5 long and narrow, without concavity, with anterior row of 21 and posterior row of six relatively short setae; median lobe strongly curved, sharply pointed in lateral view, evenly tapering, with narrowly rounded apex in ventral view; paramere with notch on its dorsal side and small, less evidently separated subdistal part with a tuft of dense setae; proximal setae inconspicuous (Fig. 10A–D). For complete description, see Balke (1998).

##### Distribution.

**Figure 12.**
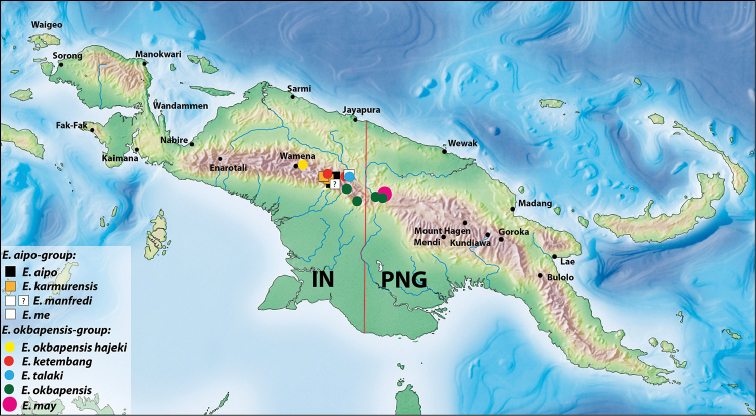
Map of New Guinea showing distribution of the species of both groups. The white square with a question mark indicates the male from Aipomek-Tanime area, which most likely belongs to *E. manfredi* (Balke, 1998).

Papua: Pegunungan Bintang Regency and PNG: Sandaun Province (Fig. 12).

#### 4. Exocelina talaki

Taxon classificationAnimaliaORDOFAMILIA

(Balke, 1998)

Figs 9 , 11

Copelatus (Papuadytes) talaki Balke, 1998: 337; Nilsson 2001: 77 (catalogue).
Papuadytes talaki (Balke, 1998): Nilsson and Fery 2006: 56 (comb.n.).
Exocelina talaki (Balke, 1998): Nilsson 2007: 34 (comb.n.).

##### Type locality.

Papua: Pegunungan Bintang Regency, Borme, ca. 04°24'S; 140°25'E, 1200 m a.s.l.

##### Type material studied.

*Holotype*: male “IRIAN JAYA: Borme ca. 140°25'E, 04°24'S 1200 m, 2.9.1993 leg. M. Balke (1)”, “HOLOTYPUS” [red], “Copelatus talaki Balke des. 1997” [red] (NHMW). *Paratypes*: Three females with the same label as the holotype and additionally with red labels “Paratypus Copelatus talaki Balke des. 1997”, one of them with two additional labels “M. Balke 3294” [green] and “M. Balke 6415” [green text] (NHMW).

##### Diagnosis.

Beetle small (TL-H 3.25–3.4 mm); oblong-oval; reddish brown to brown, with paler head and pronotum, with fine punctation and weakly impressed microreticulation, shiny; pronotum with distinct lateral bead; male antennae simple (Fig. 9); male protarsomere 4 with large, thick, strongly curved anterolateral hook-like seta; male protarsomere 5 long and narrow, without concavity, with anterior row of 14 and posterior row of seven relatively short setae; median lobe strongly curved, sharply pointed in lateral view, evenly tapering, with broadly pointed apex in ventral view; paramere with notch on its dorsal side and small, less evidently separated subdistal part with a tuft of dense setae; proximal setae inconspicuous (Fig. 11A–D). For complete description, see Balke (1998).

##### Distribution.

Papua: Pegunungan Bintang Regency (Fig. 12). The species is known only from the type material.

## Key to species of the *Exocelina aipo*- and *E. okbapensis*-groups

Since the representatives of the groups occur on the same geographic area (the central part of New Guinea), we treat them in the same key to simplify identification.

The key is based mostly on male characters. In many cases females cannot be assigned to species due to similarity of their external and internal structures (for female genitalia see figs 17a and 17b in Shaverdo et al. (2005)). Some species are rather similar in external morphology; therefore, in most cases male genitalia need to be studied for reliable species identification. Numbers in parentheses refer to the arrangement of species descriptions above.

**Table:** 

1	Male protarsomere 5 concave ventrally, shorter, usually with some ventral setae enlarged and shifted to base (Fig. 1; figs 25–28 in Balke 1998)	**2** (***aipo*-group)**
–	Male protarsomere 5 long and narrow, without concavity, setae not modified	**5** (***okbapensis*-group)**
2	Beetle distinctly larger, TL-H: 4.4–5.4 mm. Male protarsomere 5 with concavity deep but small and rounded, without enlarged ventral setae (fig. 26 in Balke 1998). Male antennae distinctly modified (fig. 9 in Balke 1998)	***karmurensis***
–	Beetle distinctly smaller, TL-H: 3.5–4.55 mm. Male protarsomere 5 with concavity large, with some ventral setae enlarged and shifted to base (as in fig. 25 in Balke 1998). Male antennomeres distinctly modified or just stout	**3**
3	Male antennomeres stout (fig. 10 in Balke 1998). Dorsal punctation stronger and denser	***manfredi***
–	Male antennae distinctly modified (figs 8, 11 in Balke 1998). Dorsal punctation weaker	**4**
4	Male antennomeres 3–6 strongly enlarged, 3–5 largest, 4 and 5 slightly rounded, 2 and 7–9 slightly enlarged (fig. 8 in Balke 1998). Male protarsomere 5 with concavity deeper (fig. 25 in Balke 1998). Dorsal punctation distinct but weak	***aipo***
–	Male antennomeres 3–7 distinctly enlarged, 3–5 largest, not rounded, 2 and 8–9 slightly enlarged (fig. 11 in Balke (1998)). Male protarsomere 5 with concavity shallower (fig. 27 in Balke (1998)). Dorsal punctation stronger and denser	*** me***
5	Male protarsomere 4 with weakly curved anterolateral “hook-like” seta, which is smaller or equal to more laterally situated large seta (Fig. 7D)	**(2) *may* sp. n.**
–	Male protarsomere 4 with large, thick, strongly curved anterolateral hook-like seta, evidently larger than more laterally situated large seta (e.g., Fig. 5D)	**6**
6	Median lobe with straight, sharply pointed apex in lateral view	**7**
–	Median lobe with apex slightly to strongly curved downwards, rounded in lateral view	**8**
7	Beetle larger, TL-H: 3.9–4.35 mm, dark brown to piceous (Fig. 8)	**(3) *ketembang***
–	Beetle smaller, TL-H: 3.25–3.4 mm, reddish brown to brown (Fig. 9)	**(4) *talaki***
8	Antennomeres 2–6 simple, in some males slightly stout (Fig. 3). Abdominal ventrite 6 with 5–16 long lateral striae on each side. Median lobe and paramere as in Fig. 5A–C	**(1) *okbapensis* sp. n.**
–	Antennomeres 2–6 slightly, but evidently enlarged in males and stout in females (Fig. 2). Abdominal ventrite 6 with 16–22 long lateral striae on each side. Median lobe and paramere as in Fig. 6A–C	**(1a) *okbapensis hajeki* ssp. n.**

## Habitats

**Figure 13.**
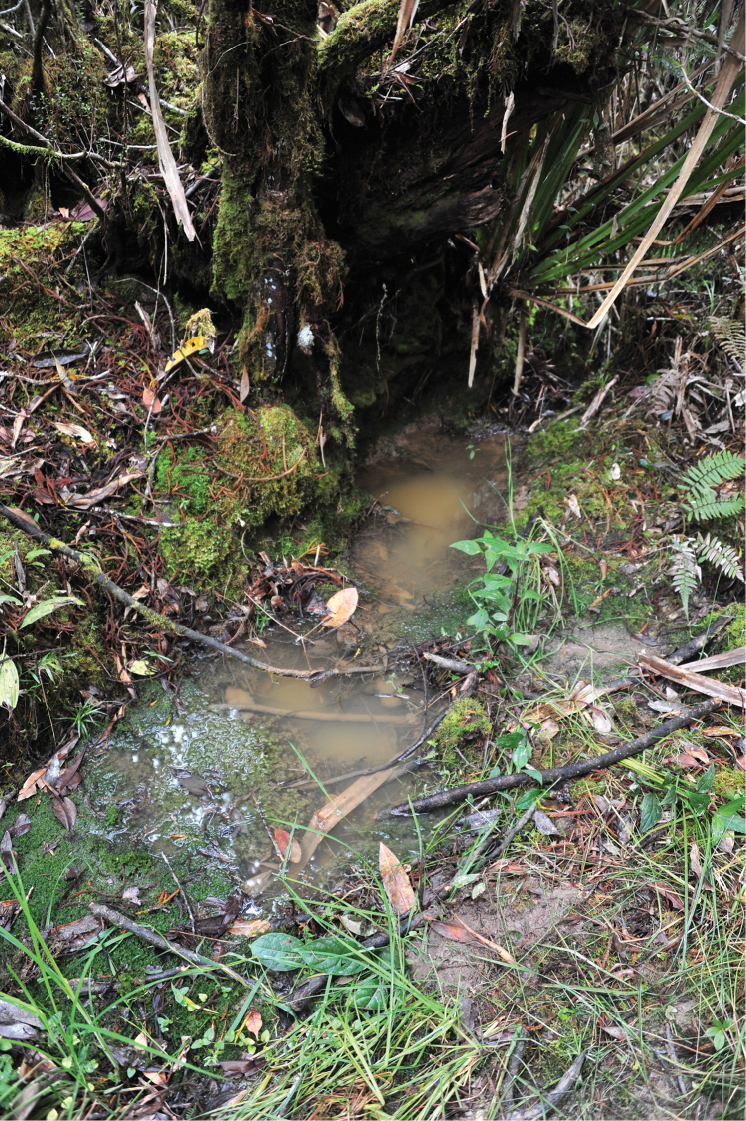
Forest puddle, habitat of *Exocelina okbapensis hajeki* ssp. n. 10 km NE Wamena, forest above ‘Baliem vall. Resort’. Photo by Jiří Hájek.

**Figure 14.**
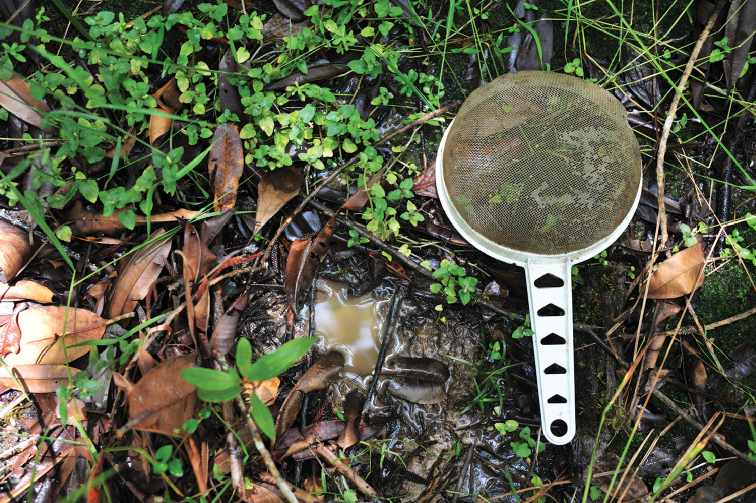
Wed forest ground, habitat of *Exocelina okbapensis hajeki* ssp. n. 10 km NE Wamena, forest above ‘Baliem vall. Resort’. Photo by Jiří Hájek.

The studied species have the same habitat preferences as those described in Shaverdo et al. (2012). They are associated with running water, but avoid the current, i.e., their preferred microhabitats are small creeks, small and quiet backflows, puddles at the edge of streams and creeks, and other similar situations (e.g., Figs 13–14). Habitats can be shadowed (in the forest) or sun exposed.

## Supplementary Material

XML Treatment for Exocelina okbapensis

XML Treatment for Exocelina okbapensis hajeki

XML Treatment for Exocelina may

XML Treatment for Exocelina ketembang

XML Treatment for Exocelina talaki
